# Redox Properties of TiO_2_ Thin Films Grown on Mesoporous Silica by Atomic Layer Deposition

**DOI:** 10.1021/acs.jpclett.3c00834

**Published:** 2023-05-12

**Authors:** Wang Ke, Xiangdong Qin, Robert M. Palomino, Juan Pablo Simonovis, Sanjaya D. Senanayake, José A. Rodriguez, Francisco Zaera

**Affiliations:** † Department of Chemistry and Center for Catalysis, University of California, Riverside, California 92521, United States; ‡ National Synchrotron Light Source II, Brookhaven National Laboratory, Upton, New York 11973, United States; § Department of Chemistry, Brookhaven National Laboratory, Upton, New York 11973, United States

## Abstract

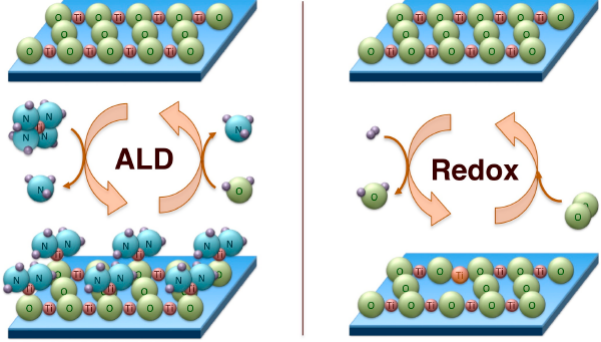

The redox properties of titania films grown by ALD on SBA-15, a silica-based mesoporous material, were characterized as a function of thickness (that is, the number of ALD cycles used). ^29^Si CP/MAS NMR helped to identify the nature of the surface species that form in the initial stages of deposition, and infrared absorption spectroscopy was used to follow the transition from silica to titania surfaces. The reducibility of the titania sites by CO and H_2_ was studied *ex situ* using EPR and *in situ* with ambient-pressure XPS. It was determined that the titania ALD films are amorphous and easier to reduce than crystalline titania and that the reduction is reversible. A transition in the nature of the surface was also observed, with unique mixed Si–O–Ti sites forming during the first few ALD cycles and a more typical titania surface progressively developing as the film grows in thickness.

Much heterogeneous catalysis relies on the use of high-surface-area porous oxide materials such as silica or alumina. Originally, these solids were viewed as a convenient way to disperse the catalytically active phase, typically a metal, as small nanoparticles in order to optimize the amount of surface exposed. However, it has repeatedly been shown that these oxide supports often participate in the promotion of the reaction, providing new catalytic sites either by themselves or at the interfaces that they form with the other phases (the metal nanoparticles, for instance). Several critical industrial processes such as oil cracking and organic synthesis (alkylation, epoxidation, hydroxylation, oximation) are promoted by porous oxides alone, and in those cases, the catalytic performance often relies on the acid–base or redox properties of the solid. It is therefore highly desirable to be able to tune such properties in a controlled fashion. One way in which this can be accomplished is by varying the composition of bulk mixed oxides during their synthesis, as is commonly done with zeolites and other aluminosilicates. Alternatively, it may be possible to tune the nature of mixed-oxide catalytic sites by modifying single-component porous oxides via the controlled deposition of a second element on their surfaces. One promising approach to carrying out such surface modification is by using atomic layer deposition (ALD).

ALD, a chemical procedure designed to deposit thin films conformally and with submonolayer thickness control,^1−4^ has acquired some prominence in recent years as a useful synthetic method for the preparation of heterogeneous catalysts with specific characteristics.^2,3,5,6^ One proven application of ALD is as a way to protect the active phase of catalysts in order to avoid sintering, leaching, or coking.^7−13^ ALD can also be used to cover an inert support with a thin layer of a catalytically active oxide to add stability^14−18^ or to create confined environments.^19^ Finally, ALD has been tested for the creation of thin layers of a new active material (Nb_2_O_5_ or VO_*x*_, for instance) inside the surface of the pores of a support to control coverage^20,21^ or dispersion^22−24^ and also to add specific sites such as acidic centers.^20,25−27^ Here we report on the controlled growth of titania films on the surfaces of a silica mesoporous material, SBA-15, in order to create new mixed-oxide sites with unique redox properties.

We have already reported some results from our research on the ALD of titania films on SBA-15 supports using tetrakis(dimethylamido)Ti(IV) (TDMAT) and deionized water.^28,29^ A systematic characterization of the nature of the resulting samples, here referred to as *x* ALD-TiO_2_/SBA-15, was performed as a function of the number of ALD cycles (*x*) using a combination of techniques, including N_2_ adsorption–desorption isothermal measurements, electron microscopy, transmission Fourier transform infrared (IR) spectroscopy, UV/visible spectroscopy, X-ray absorption spectroscopy (XAS, both XANES and EXAFS), inductively coupled plasma atomic emission spectroscopy (ICP-AES), X-ray photoelectron spectroscopy (XPS), X-ray diffraction (XRD), and solid-state ^29^Si cross-polarization magic angle spinning nuclear magnetic resonance spectroscopy (^29^Si CP/MAS NMR), and the experimental data were combined with density functional theory (DFT) calculations. It was found that the films grow uniformly in a layer-by-layer fashion all throughout the entire length of the pores of SBA-15, as desired, at a rate of 1.15 ± 0.05 Å/cycle. Surprisingly, though, those films exhibit quite low densities in the early stages of the deposition, about one-quarter of that of bulk titanium oxide. (They do approach the density of bulk TiO_2_ after approximately 10 ALD cycles.) The films grow via the formation of individual tetrahedral Ti–oxide units on Si–OH surface groups but exhibit unusually long Ti–O bonds, and they are amorphous, at least in the early stages of the deposition.

Figure 1 shows new representative ^29^Si CP/MAS NMR data illustrating the evolution of the different types of ALD nucleation sites present on the silica surface as the titania film is deposited. Initial reactivity takes place at both isolated (Q_3_, 102 ppm) and geminal (Q_2_, 92 ppm) silanol sites. It would appear that the geminal sites react first, as their surface coverage is seen to decrease after only 4 TiO_2_ ALD cycles. However, if the TDMAT precursor reacts with only one of the silanol groups in those sites, then new isolated OH groups would be generated; this is perhaps the explanation for the slight increase in the relative intensity of the Q_3_ peaks in the ^29^Si CP/MAS NMR spectra seen in Figure 1 for *x* = 2 and 4. What is clear is that the titania films nucleate at both the isolated and geminal silanol sites. Interestingly, some surface silanol groups appear not to be available for ALD nucleation, since some free Si–OH groups are detected by ^29^Si CP/MAS NMR even after 10 TiO_2_ ALD cycles.

**Figure 1:**
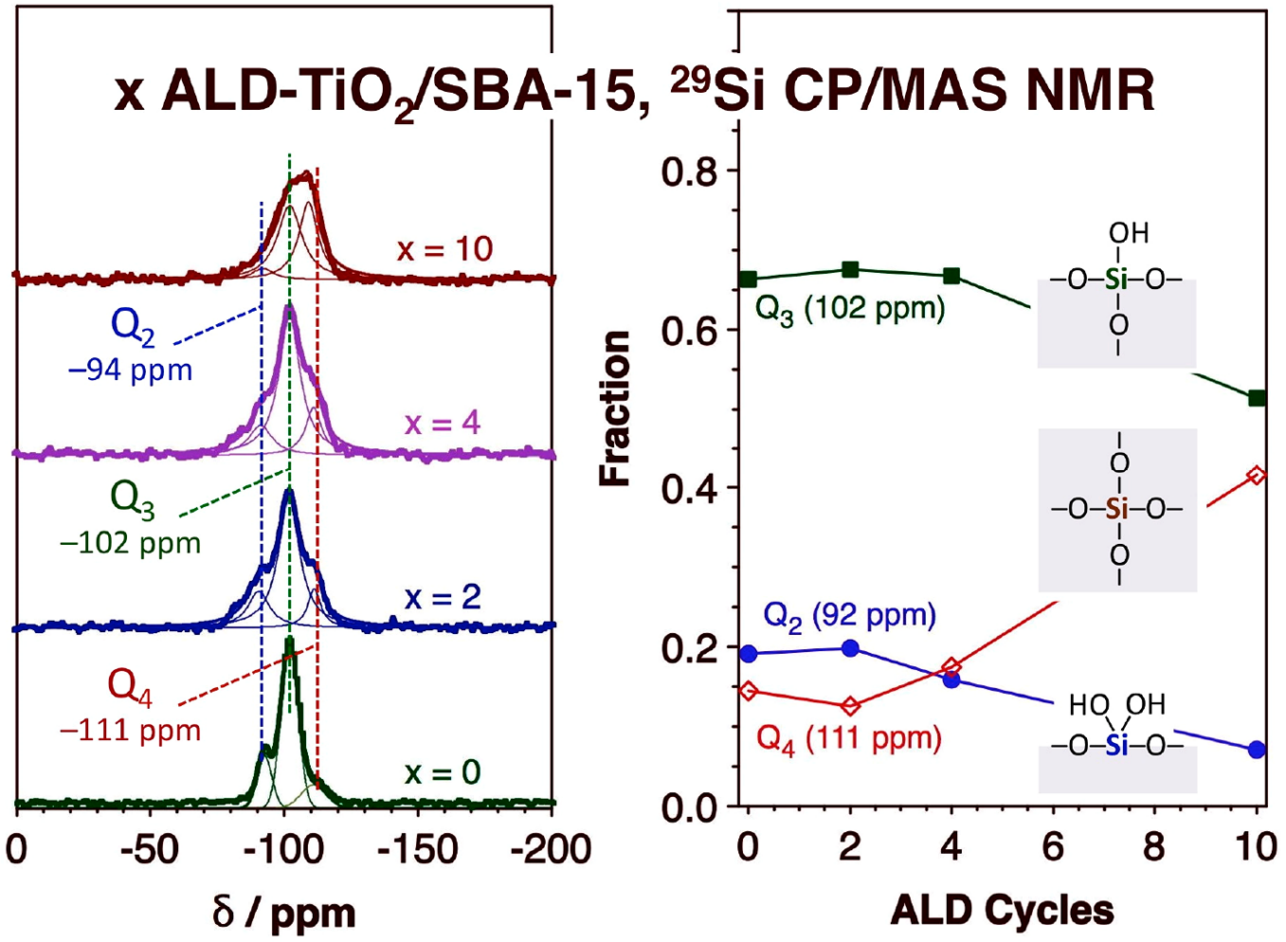
^29^Si CP/MAS NMR of SBA-15 samples after the ALD of titania films. Left: Spectra as a function of the number of ALD cycles used (from bottom to top: *x* = 0, 2, 4, and 10). The raw data are plotted as dots, while the thin lines correspond to Gaussian peaks fitted to the data. Right: Relative peak areas for the three main components of the spectra, namely, the so-called Q_2_ (92 ppm, blue filled circles), Q_3_ (102 ppm, green filled squares), and Q_4_ (111 ppm, red open diamonds) features associated with geminal silanol groups, isolated silanol groups, and Si atoms in the SiO_2_ bulk, respectively.^30−32^

The evolution of the surface silanol groups on the surface was also followed by IR, both directly, recording the changes in the peak corresponding to the O–H stretching mode of the surface nucleation sites, and indirectly, using carbon monoxide as a probe molecule and analyzing the changes in the frequency of the C–O stretching mode as a way to identify the silica- and titania-based OH surface groups.^29^Figure 2 displays some of the IR spectra obtained in the latter studies for titania films of various thicknesses, after *x* = 0, 1, 2, 3, and 4 TiO_2_ ALD cycles. The surfaces of the resulting mesoporous samples were first saturated with carbon monoxide at low temperature (125 K) and then slowly heated as the IR spectra were recorded at 10 K intervals. It can be seen in Figure 2 that some adsorbed CO is detected at low temperatures on all surfaces. Nevertheless, a clear transition is detected: whereas a peak at 2155 cm^–1^, accompanied by a smaller feature at 2137 cm^–1^, is detected with pure SBA-15, three new peaks are seen with the sample obtained after 4 TiO_2_ ALD cycles, at 2190 (main feature), 2159, and 2139 cm^–1^. The two sets of peaks can be easily assigned to CO adsorption on silica^33^ and titania^34−36^ surfaces, respectively. This conversion takes place gradually, as indicated by the progression of the intensities of the main peaks for CO adsorbed on silica (2155 cm^–1^) and titania (2190 cm^–1^) sites (Figure 3, left panel), and is roughly complete after 3 ± 1 TiO_2_ ALD cycles, at which point most of the available silanol nucleation sites have reacted with the titanium ALD precursor and the first titania monolayer has reached saturation.

**Figure 2:**
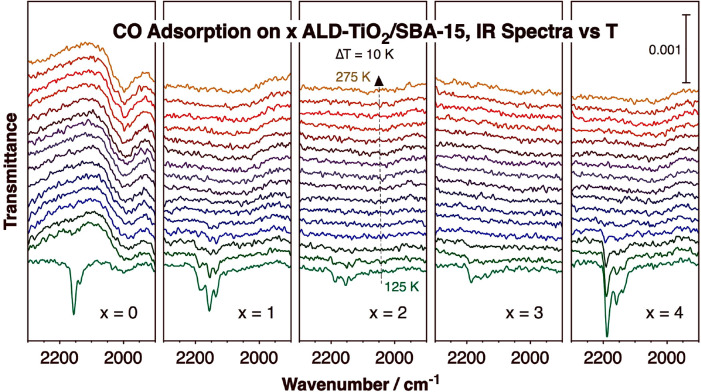
C–O stretching region of the IR spectra of carbon monoxide adsorbed on SBA-15 to which thin TiO_2_ films were added via ALD. Five panels are provided for samples treated with (from left to right) *x* = 0 (pure SBA-15), 1, 2, 3, and 4 TiO_2_ ALD cycles. In each case, the sample was first exposed to 20 Torr CO for 10 min at 125 K, after which the IR cell was evacuated and the temperature was ramped as IR spectra were acquired, in 10 K intervals.

**Figure 3:**
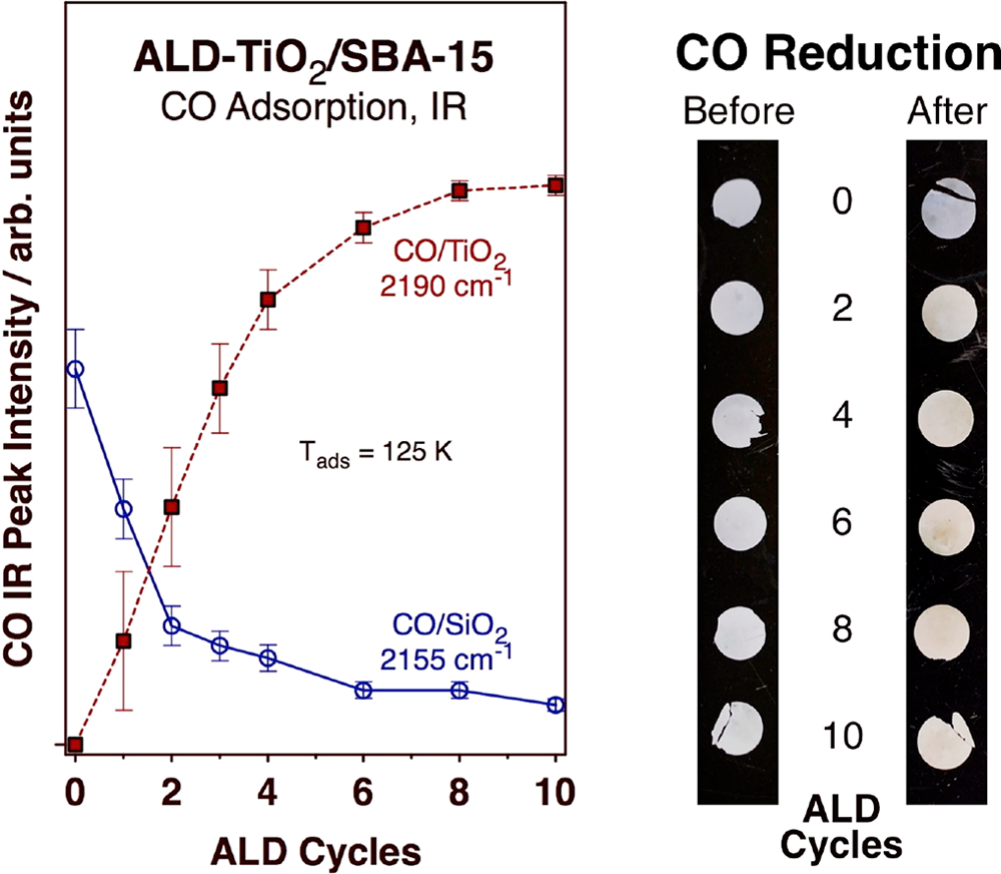
Left: Intensities of the IR peaks associated with CO adsorption on silica (2155 cm^–1^, blue open circles) and on titania (2190 cm^–1^, red solid squares) sites within the *x* ALD-TiO_2_ SBA-15 treated surfaces at 125 K as a function of the number of ALD cycles used (*x*), extracted from the data in Figure 2. Right: Pictures of the *x* ALD-TiO_2_/SBA-15 wafers used in the IR experiments before (left) and after (right) exposure to CO and temperature ramping (up to 475 K) for the samples prepared using *x* = 0, 2, 4, 6, 8, and 10 TiO_2_ ALD cycles.

Also to note from the data in Figure 2 is the fact that CO is only weakly adsorbed on all of these surfaces and that it is removed from the surface readily upon heating of the solids. However, while on pure SBA-15 most adsorbed CO is gone by 135 K, on the 4 ALD-TiO_2_/SBA-15 sample the maximum desorption rate is reached at slightly higher temperatures, about 140 K. Using Redhead’s analysis,^37^ an estimated value of *A* = 1 × 10^15^ s^–1^ for the preexponential factor, and a heating rate of 2 K/min, the activation energies of the process that removes CO from the surfaces of the two extreme cases in Figure 2, namely, for *x* = 0 and 4, are estimated to be *E*_a_ ≈ 42 and 46 kJ/mol, respectively. It would be tempting to associate those values with the heat of desorption of CO from the surface, but it needs to be remembered that carbon monoxide is a strong reducing agent and that the titanium ions in titania can be converted from a +4 to a +3 oxidation state relatively easily as oxygen atoms are removed from the titania lattice. Visual evidence for this reaction in our case is provided by the pictures of our samples before versus after exposure to CO and heat (Figure 3, right panel): the darker color seen to develop upon such treatment indicates the creation of color centers from the reduction of the titania films. It is also observed that the thicker the films, that is, the more TiO_2_ ALD cycles that are used, the darker the solids become upon treatment with CO. It is this ease with which the amorphous titania films grown on SBA-15 by ALD can undergo redox interconversion that we highlight in the present study.

More direct evidence of the reducibility of our samples was acquired by electron paramagnetic resonance spectroscopy (EPR). Figure 4 displays representative results from such studies for the 4 ALD-TiO_2_/SBA-15 solid. A general survey of the behavior of that sample after treatment with reducing and oxidizing atmospheres is provided in the left panel. As expected, pure SBA-15 shows no EPR signal (bottom, green trace), since the Si^4+^ in SiO_2_ has no unpaired electrons. Moreover, the EPR of the freshly deposited TiO_2_ film is also flat (second from bottom, purple), indicating that the ALD titania films are fully oxidized and that all titanium ions are in their +4 oxidation state. On the other hand, upon exposure to a H_2_ atmosphere at 725 K, a large symmetrical peak develops, centered at 3331.7 G (*g* = 1.994; second from top, blue trace), clearly indicating the partial reduction of the titania film. The value of *g* obtained here is lower than those reported for most crystalline bulk titania samples, but it is within the range of what has been seen for submonolayer coverages of TiO_2_ on MCM-48 (another silica mesoporous material; *g* = 1.932)^38^ and not far from values obtained with amorphous TiO_2_ (*g* = 2.003)^39^ and with PVP-capped TiO_2_ nanoparticles (*g* = 2.005).^40^ Our results are consistent with the nature of our sample, namely, a surface TiO_2_ film that is both amorphous and thin. Finally, exposure of the sample to O_2_ at 725 K (Figure 4, left panel top, red trace) reoxidizes most (if not all) of the Ti^3+^ centers made by the reduction treatment. Interestingly, in addition to a small residual peak from the Ti^3+^ left over, a second small feature is also seen in this case at 3321 G (*g* = 2.001) likely due to the presence of a few surface O^–^ species.^38^

**Figure 4:**
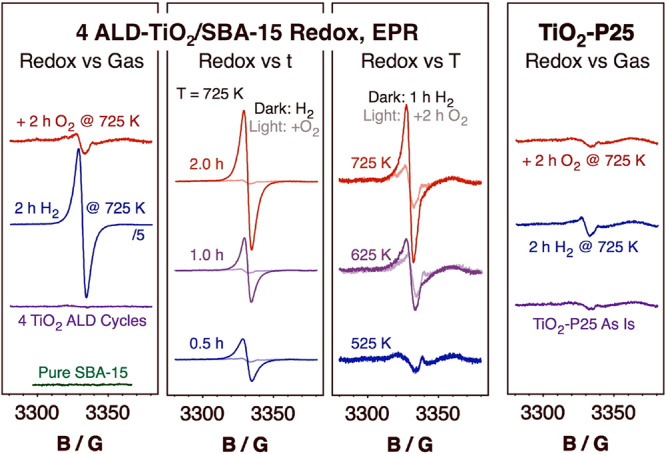
EPR spectra of SBA-15 mesoporous materials treated with 4 cycles of TiO_2_ ALD after a variety of treatments. Left panel: From bottom to top, pure SBA-15 (green trace), and after sequential TiO_2_ deposition (purple), 2 h exposure to H_2_ at 725 K (blue) and 2 h exposure to O_2_ at 725 K (red). Second from left: 4 ALD-TiO_2_/SBA-15 after exposure to either H_2_ (dark lines) or O_2_ (light lines) atmospheres at 725 K as a function of time. Second from right: 4 ALD-TiO_2_/SBA-15 after either 1 h H_2_ (dark lines) or 2 h O_2_ (light lines) exposures as a function of temperature. Right: Reference data for crystalline Degussa TiO_2_-P25, taken as is (bottom, purple line) and after a sequential 2 h of H_2_ (middle, blue) and 2 h of O_2_ (top, red) treatments at 725 K. The *y*-axis scale is plotted in arbitrary units, but it is the same in all four panels.

Additional EPR kinetic data for the oxidation and reduction processes are provided in the two central panels of Figure 4. The second panel shows the evolution of the EPR peak as a function of treatment time at 725 K, whereas the third panel displays the progress seen after a fixed interval of time at different temperatures. It is clear that under H_2_ atmospheres both longer times and higher temperatures lead to the formation of more reduced titanium sites (dark traces). Moreover, given that the signal growth appears to be approximately linear with reaction time, it would seem that the film reduction had not reached its limit in any of the experiments reported here. The reduced films could be reoxidized via exposure to O_2_ (light traces), but the effectiveness of that step appears to be diminished at higher temperatures. On the whole, though, it is clear that these *x* ALD-TiO_2_/SBA-15 thin films can be easily and extensively reduced and that the reduction is by and large reversible. Although we have no way to calibrate our signals to determine the absolute fraction of Ti ions that become reduced, we can safely say that it is much larger than what can be obtained with crystalline samples: the right panel of Figure 4, which reports similar EPR data collected with a Degussa TiO_2_–P25 powder (a well-known ∼70–80% anatase + 30–20% rutile crystalline mix), clearly shows much less Ti^3+^ generation.

Finally, the reversible redox properties of the ALD titania films were tested *in situ* in the presence of H_2_ and O_2_ atmospheres by AP-XPS. Typical data obtained in the O 1s XPS region for the samples prepared using *x* = 2 (left) and *x* = 4 (right) TiO_2_ ALD cycles are shown in Figure 5. Two peaks could be identified in these spectra: the first, centered at a constant binding energy (BE) of 532.75 eV, associated with the silica substrate, and a second at approximately 2 eV lower BE that originates from the growing titania film.^29^ Interestingly, the exact position of the latter feature changes depending on the conditions used, namely, on the gas to which the solid is exposed and on temperature. When exposing the as-prepared samples to a H_2_ atmosphere at 300 K, the O 1s XPS peak corresponding to titania is centered at BE = 530.6 ± 0.1 eV (bottom, blue traces), a value that we associate with fully oxidized titanium ions in their Ti^4+^ state. However, upon increasing the temperature to 500 K, that peak blue-shifts to values of about BE = 531.0 ± 0.1 eV (middle, purple). This, we believe, provides evidence for a partial reduction leading to the formation of some Ti^3+^ centers. Further treatment at 500 K in an O_2_ atmosphere reverses the reduction, as the corresponding O 1s XPS peak returns to a value of BE = 530.7 ± 0.1 eV. The *in situ* AP-XPS data are therefore consistent with the results from the *ex situ* EPR studies discussed above (Figure 4), in that both attest to the easy and reversible redox behavior of the thin TiO_2_ ALD films. Unfortunately, quantitation of the fraction of oxygen vacancies and Ti^3+^ centers created by the H_2_ treatment and reverting to TiO_2_ upon O_2_ exposure (both at 500 K) using these XPS data is difficult, as it appears to be within the experimental error of the measurements: it does seem that for the 4-ALD-cycles case the O 1s XPS component due to the titania films in Figure 5 grows by about 7% of the total O 1s XPS area (∼50% of the titania component) upon oxidation of the reduced film, but this effect is less evident in the 2-ALD-cycles case.

**Figure 5:**
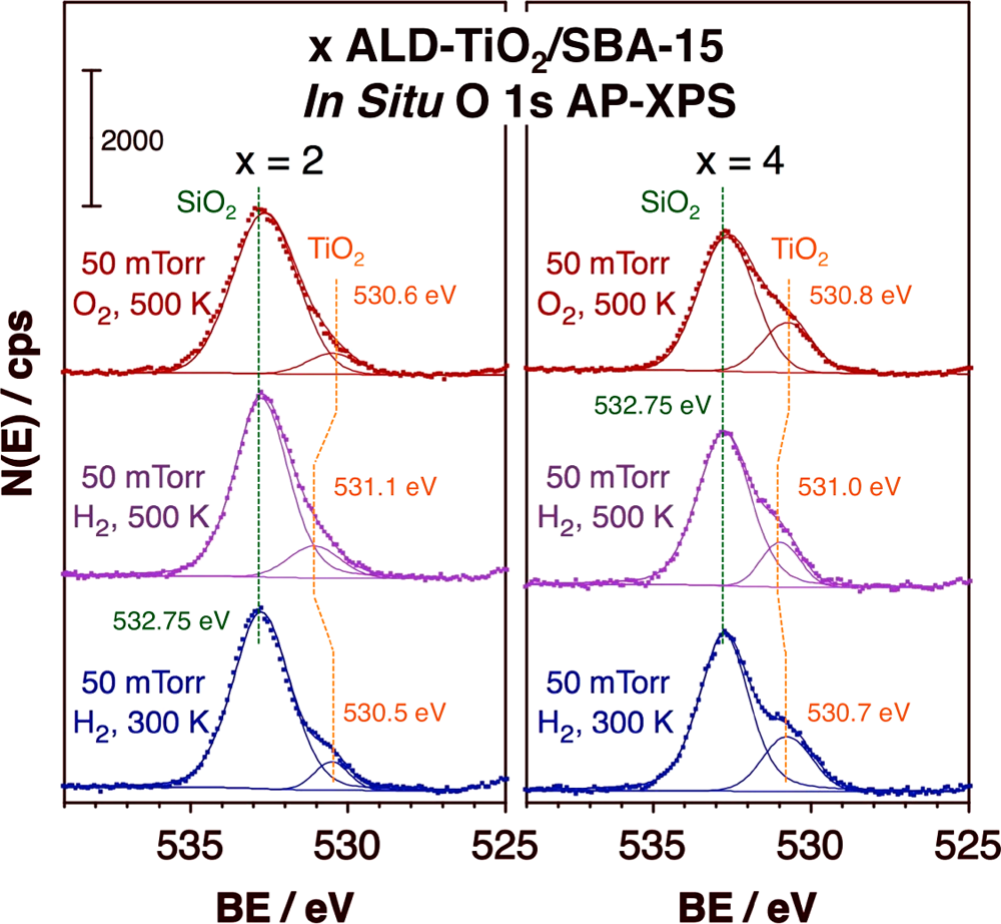
*In situ* O 1s AP-XPS data for SBA-15 mesoporous solids treated with either 2 (left) or 4 (right) cycles of TiO_2_ ALD under different atmospheres. In each panel, three sets of data are shown, for the material under 50 mTorr H_2_ at 300 K (bottom, blue), 50 mTorr H_2_ at 500 K (middle, purple), and 50 mTorr O_2_ at 500 K (top, red). The raw data are plotted as dots, and fits to two Gaussian peaks, associated with SiO_2_ (BE = 532.75 eV) and TiO_2_ (BE = 530.5 to 531.1 eV), are shown as thin solid lines.

A systematic AP-XPS study of this redox behavior was carried out with all of the *x* ALD-TiO_2_/SBA-15 samples as a function of gas (H_2_ or O_2_) and temperature (300 to 500 K). The O 1s AP-XPS spectra recorded for all samples exposed to 50 mTorr H_2_ at three temperatures (300, 400, and 500 K) are displayed in Figure S1 (Supporting Information), and a summary of the information extracted from those data in terms of peak areas and peak positions is provided in Figure 6. In terms of peak areas (Figure 6, left panel), it is seen that the area of the feature due to SiO_2_ (BE = 532.75 eV) decreases in a linear fashion as a function of the number of ALD cycles used (*x*), at the expense of the linear growth of the peak component due to TiO_2_ (BE = 530.6 eV); only in going from 8 to 10 cycles do those trends deviate from linearity, as the titania films become thicker. Two observations are worth highlighting here: (1) the total O 1s XPS signal intensity decreases with increasing number of TiO_2_ ALD cycles, as already reported by us and explained by a lower oxygen atomic density in the TiO_2_ films compared to that in the SiO_2_ substrate;^29^ and (2) the areas of the two O 1s XPS components, for SiO_2_ and TiO_2_, remain approximately constant in each sample as the reducing temperature is increased from 300 to 500 K. The deposition rate that can be extracted from these data is approximately 0.95 ± 0.20 Å/cycle, not far from that estimated using the data from N_2_ adsorption–desorption isotherms.^29^

**Figure 6:**
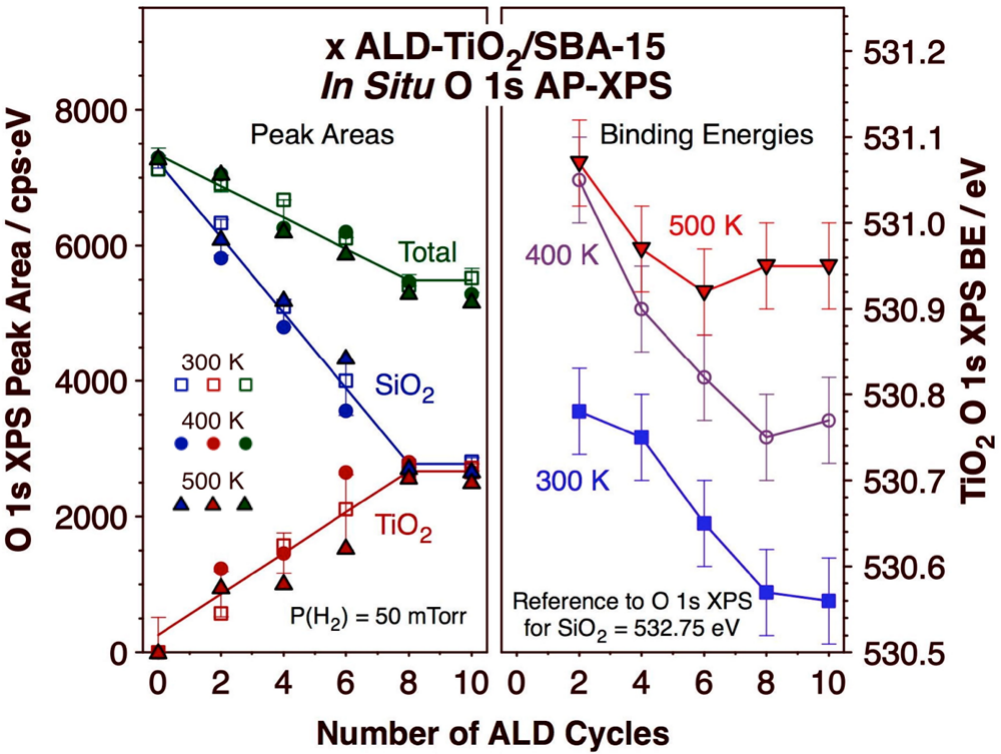
Summary of the data extracted from the *in situ* O 1s AP-XPS data taken for the *x* ALD-TiO_2_/SBA-15 samples while under a 50 mTorr H_2_ atmosphere as a function of the number of ALD cycles used (*x*). Left panel: Peak areas for the components associated with SiO_2_ and TiO_2_, and total area. Right panel: Binding energies (BEs) for the peak associated with TiO_2_. Data are reported for three different temperatures: 300, 400, and 500 K. The raw O 1s AP-XPS data are shown in Figure S1.

The right panel of Figure 6 provides information on the shifts that the O 1s XPS component due to the TiO_2_ films undergoes when under a H_2_ atmosphere as a function of temperature, for all film thicknesses (that is, all values of ALD cycles, *x*). For the starting materials, right after preparation and when under 50 mTorr H_2_ at 300 K, the BE of that peak displays the highest value, BE ≈ 530.78 eV, for the thinnest (*x* = 2) film, and decreases monotonically with film thickness until reaching a value of BE = 530.56 eV for *x* = 10 (blue filled squares; two significant figures are provided here after the decimal point because a higher peak position accuracy can be extracted from the fit of the raw data to Gaussian peaks). We suggest that the higher BE values for the O 1s XPS peak of the thinner films may reflect the formation of mixed Si–O–Ti surface sites, since the electronegativity of Si (1.90) is higher than that of Ti (1.54); as the film becomes thicker, that contribution is minimized. Alternatively, the observed trend may indicate a more extensive coordination of the titanium ions to terminal OH groups in the thinner films, on average, as the electronegativity of H (2.2) is even higher than that of Si. In any case, a systematic blue shift of approximately 0.2 eV is seen with all of the samples upon an increase in the temperature to 400 K (Figure 6, right panel, purple open circles), a change associated with the reduction of some Ti ions to a +3 oxidation state. Further heating to 500 K continues this trend, more strongly with the thicker films; it does appear that the film reduction may be incomplete in some of the thicker films at 400 K. Ultimately, the BE of the O 1s XPS TiO_2_ component in most of the TiO_2_ films approaches a final value of BE = 530.95 ± 0.05.

The corresponding Si 2p and Ti 2p AP-XPS spectra for the same samples and conditions as those reported above are provided in Figures S2 and S3, respectively (Supporting Information), and a summary of the parameters extracted from those data is reported in Figure 7. The peak areas (Figure 7, left panel) follow the same qualitative trends seen for the O 1s XPS signals in Figure 6, namely, the signal intensity of the Si 2p XPS peaks decreases monotonically, in an approximately linear fashion, with the number of ALD cycles used to grow the titania film (*x*) as the corresponding Ti 2p XPS peak area grows. These areas are reported in Figure 7 in relative arbitrary units but were corrected by the appropriate energy analyzer sensitivities to the dfferent elements^41^ to extract information on surface atomic composition. On that basis, it is clear from the data in Figure 7 that the atomic density of Ti in the titania films is much lower than that of the Si atoms in the underlying SBA-15 substrate, as already concluded on the basis of the evolution of the O 1s XPS peak areas for SiO_2_ and TiO_2_ shown in Figure 6. This was reported before and justified in terms of the open structure that forms on the surface during the first stages of the titania film growth.^29^

**Figure 7:**
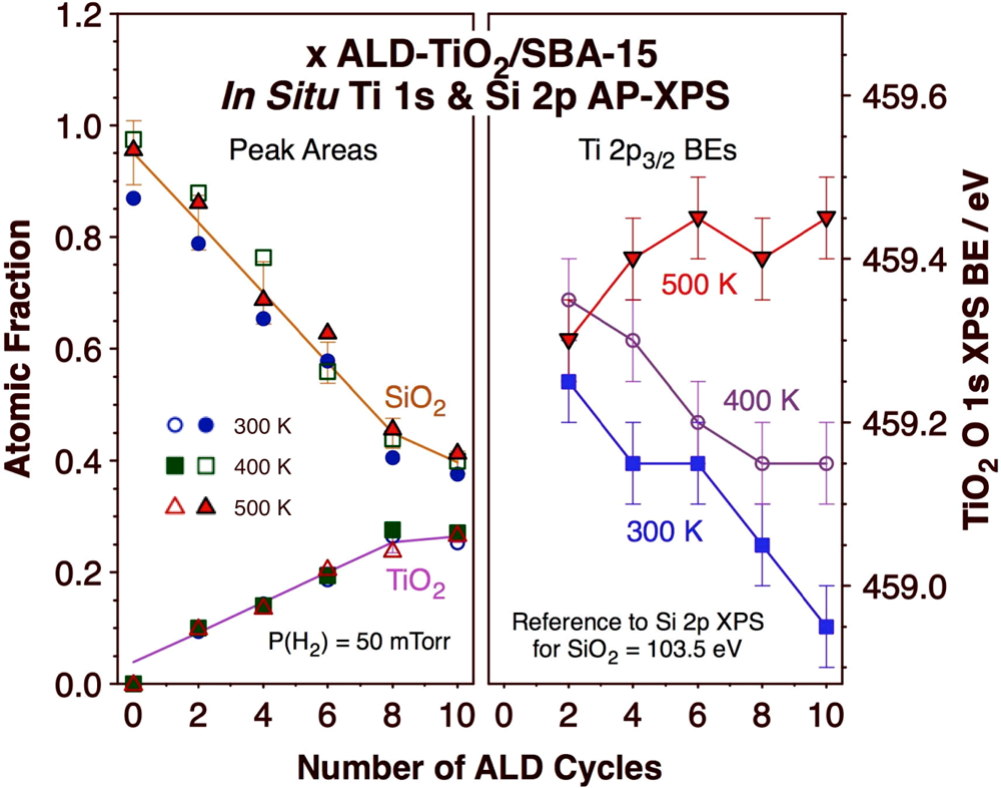
Summary of the data extracted from the *in situ* Si 2p and Ti 2p AP-XPS data taken for the ALD-TiO_2_/SBA-15 samples while under a 50 mTorr H_2_ atmosphere as a function of the number of ALD cycles used (*x*). Left panel: Peak areas. Right panel: BEs for the Ti 2p_3/2_ peaks. Data are reported for three different temperatures: 300, 400, and 500 K. The raw Si 2p and Ti 2p AP-XPS data are shown in Figures S2 and S3 (Supporting Information).

The right panel of Figure 7 also looks qualitatively similar to the right panel of Figure 6. Indeed, in both, it can be seen that the BE (of the Ti 2p_3/2_ XPS peak in Figure 7) decreases with increasing number of TiO_2_ ALD cycles *x* (for the 300 and 400 K cases at least) and increases with increasing temperature for each sample. It would appear that the titanium atoms are more oxidized in the initial stages of the ALD deposition, a fact that we again explain on the basis of a higher average coordination number to more electronegative O–Si and/or O–H surface groups; those are progressively replaced by Ti–O–Ti bonds as the titania film network grows past the first monolayer. The evolution of the Ti 2p BE as a function of temperature in the presence of the H_2_ atmosphere, a reducing environment, is more difficult to explain, as the expected reduction of the titanium ions at higher temperatures should yield lower, not higher, BEs. It should be remembered, however, that the values of the XPS BEs depend not only on the oxidation state but also on the overall electronic environment around the probed atom. In this case, the newly formed Ti^3+^ ions after reduction at 400 and 500 K may be affected to a higher degree than the original Ti^4+^ ions (before reduction) by the surrounding Si atoms and by the OH surface groups. What is clear is that the trends match those seen with the O 1s XPS peaks.

In summary, the data reported here clearly show that titania films grown by ALD on silica (SBA-15) supports are easily reduced under CO or H_2_ atmospheres and are reversibly reoxidized upon treatment with O_2_. This behavior may be explained at least in part by the amorphous nature of the films but may also be influenced by the formation of new Si–O–Ti mixed oxide surface sites. The newly formed sites may be of great relevance to catalysis, as many chemical conversions require the promotion of redox steps. Titania is used extensively in catalysis for this purpose, by itself, in photocatalysis^42−44^ or electrocatalysis,^45−48^ or in conjunction with metals to thermally catalyze selective oxidation steps.^49−52^ The ALD methodology advanced here provides a way of tuning the redox properties of those sites in a more controlled fashion than by adjusting the stoichiometries of mixed oxides during their synthesis. Past synthetic methods attempting to produce titania surfaces with specific catalytic properties have included the use of a reducing gas,^53^ calcination,^54,55^ laser irradiation,^56,57^ plasmas,^58^ high-energy particle bombardment,^59^ and specific chemical synthetic strategies,^60−67^ but none of those have led to the development of protocols where such properties can be tuned in a systematic manner. Here we advance the notion that postmodification of surfaces via ALD perhaps provides a better way to do this. The method is also quite versatile and general, and can be easily extended to the synthesis of catalysts based on other reducible oxides (ceria, zirconia, hafnia)^5,68^ or even to the making of mixed oxides (silica–alumina)^27^ with unique acid–base properties.

## Supporting Information Available

Experimental details; *in situ* O 1s, Si 2p, and Ti 2p AP-XPS data, taken in the presence of 50 mTorr H_2_, versus the number of TiO_2_ ALD cycles and temperature (PDF)
