# Supplementary material for: Redox Properties of TiO2 Thin Films Grown on Mesoporous Silica by Atomic Layer Deposition
Source: J Phys Chem Lett. 2023 May 12;14(20):4696–703. doi: 10.1021/acs.jpclett.3c00834 (PMC10226118; doi:10.1021/acs.jpclett.3c00834)
Supplement: Supplementary file 1 — jz3c00834_si_001.pdf [file jz3c00834_si_001.pdf]

Redox Properties of TiO_2 Thin Films Grown on Mesoporous Silica by Atomic Layer Deposition

Wang Ke,[1,∞] Xiangdong Qin,[1,‡] Robert M. Palomino,[2,fi] Juan Pablo Simonovis,[2,§] Sanjaya D. Senanayake,[3] José A. Rodriguez,[3] Francisco Zaera[1,*]

[1] Department of Chemistry and Center for Catalysis, University of California, Riverside, CA 92521, USA

[2] National Synchrotron Light Source II, Brookhaven National Laboratory, Upton, NY 11973, USA

[3] Department of Chemistry, Brookhaven National Laboratory, Upton, NY 11973, USA

[*] Corresponding Author. Email: zaera@ucr.edu

Supporting Information

1. Experimental Details 2
2. Figure S1 5
3. Figure S2 6
4. Figure S3 7
5. References 8

1. Experimental Details

The titanium oxide films were deposited on commercial SBA-15 (ACS Material) using a homemade ALD reactor described in detail elsewhere,[1] by alternating exposures to tetrakis(dimethylamido)Ti(IV) (TDMAT, Aldrich-Sigma, 99.999% purity in a trace metals basis) and deionized water at 375 K, purging with pure N_2 in between. More details of the ALD process and the extensive characterization of these samples was already reported in previous publications.[1,2] Five samples were prepared, using x = 2, 4, 6, 8, and 10 ALD cycles, to systematically test the effect of TiO_2 film thickness on redox behavior. They are referred here as x ALD-TiO_2/SBA-15, where x is the number of ALD cycles used.

Ex situ solid-state ^{29}Si cross polarization magic angle spinning nuclear magnetic resonance (CP/MAS-NMR) spectra were acquired on a Bruker Avance 600 spectrometer, employing a cross-polarization contact time of 2 ms, a ^1H decoupling bandwidth of 80 kHz, and a recycle time of 3 s. Data were acquired as 12,000 co-added 2,048 complex data point FIDs with a 100 kHz sweep width. Post acquisition processing consisted of exponential multiplication with 200 Hz of line broadening and zero filling to 4,096 data points. Chemical shifts were referenced to an external DSS sample.

In situ transmission infrared spectroscopy (IR) characterization experiments were performed on a Bruker Tensor 27 Fourier transform infrared (FTIR) spectrometer equipped with a deuterated triglycine sulfate (DTGS) detector. About 10 mg of the catalyst was pressed into a self-supporting wafer and loaded inside a homemade quartz cell with NaCl windows.[3] The cell was

evacuated and cooled down to 125 K (using liquid nitrogen), and IR spectra of the bare samples were acquired. Afterward, those samples were exposed to 20 Torr of CO (Matheson Tri-Gas, ≥99.9% purity) for 10 min, and the cell was evacuated for 10 min. IR spectra were recorded from 125 to 475 K at 10 K intervals as the sample and cell were warmed up at a rate of 2 K/min, and corrected using background spectra obtained under the same condition before adsorption.

Ex situ electron paramagnetic resonance (EPR, also known as electron spin resonance –ESR–) analysis was carried out using a Bruker EMX EPR spectrometer equipped with a Bruker ER 4102ST cavity operating at a frequency of 9.298 GHz. Approximately 1 mg of sample was placed in a capillary glass tube, which was sealed with parafilm and placed in the cavity for analysis. The center field was set as 3330 G, and the sweep width was set as 100 G. The EPR measurements were performed at 100 K.

In situ ambient-pressure X-ray absorption spectroscopy (AP-XPS) data were collected using a commercial system (SPECS Surface Nano Analysis GmbH, Germany). The instrument is equipped with a hemispherical electron-energy analyzer (PHOIBOS 150 NAP), a dual anode Mg/Al X-ray source (from which the K_α emission from the Mg anode (hν = 1253.6 eV) was used), and standard surface preparation equipment. This instrument is capable of performing XPS measurements under gas pressures of up to approximately 1 Torr. For that to be possible, the electron energy analyzer is differentially pumped in two stages, with the entrance cone at the inlet of the first lens having an aperture 300 μm in diameter. The first stage of pumping is equipped with a residual gas analyzer (RGA) to sample any reaction products coming from the surface. The raw XPS data were fit to Gaussian peaks after subtraction of a Shirley background

in order to extract peak positions and peak intensities. The peak positions were referenced to a binding energy value of BE = 130.5 ± 0.1 eV for the Si 2p peak in SiO_2.[4]

2. Figure S1

In situ O 1s AP-XPS data for samples prepared by TiO_2 ALD on SBA-15, taken under an atmosphere of 50 mTorr of H_2. The samples correspond to several numbers of TiO_2 ALD cycles, namely, (from left to right): x = 0, 2, 4, 6, 8, and 10. Three sets of data are shown in each panel, for three temperatures: 300 K (bottom, blue traces), 400 K (middle, purple), and 500 K (top, red). The raw data, shown as dot, were fitted to two Gaussian peaks (thin lines), corresponding to the oxygen atoms associated with the SiO_2 support (BE = 532.75 eV, green vertical lines) and the TiO_2 film (BE ~ 530.6 eV, orange).

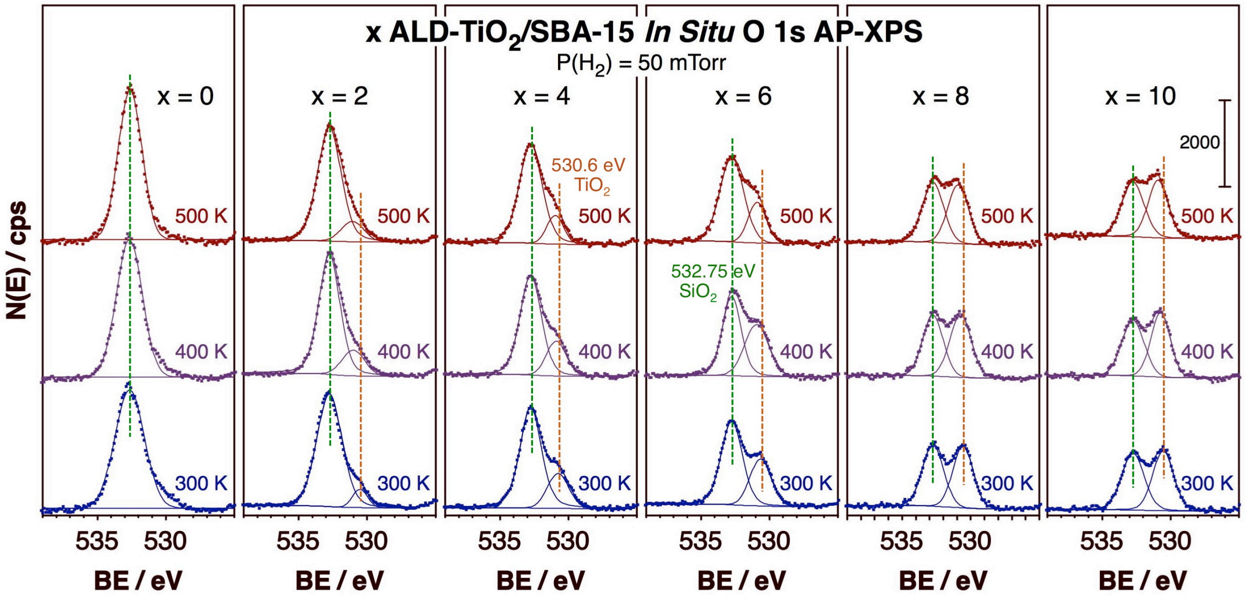

3. Figure S2

In situ Si 2p AP-XPS data for samples prepared by TiO_2 ALD on SBA-15, taken under an atmosphere of 50 mTorr of H_2. The samples correspond to several numbers of TiO_2 ALD cycles, namely (from left to right): x = 0, 2, 4, 6, 8, and 10. Three sets of data are shown in each panel, for three temperatures: 300 K (bottom, blue traces), 400 K (middle, purple), and 500 K (top, red). All peaks correspond to Si atoms in the SiO_2 (SBA-15) material, and are centered at a value of BE = 103.5 eV, used to calibrate the BE scale.

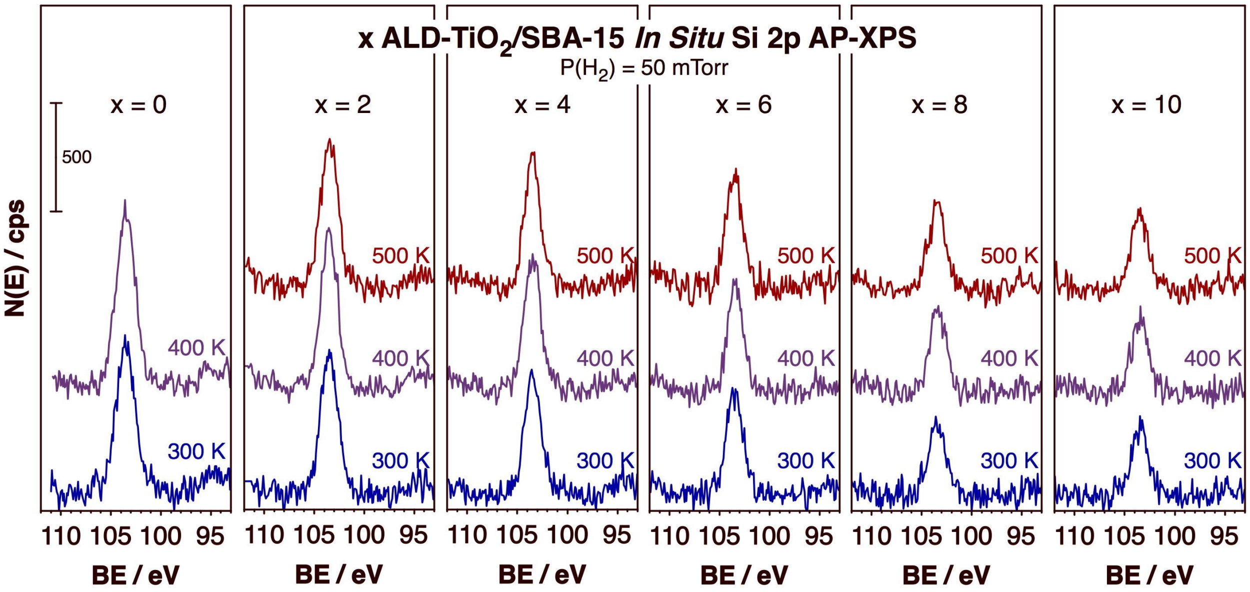

4. Figure S3

In situ Ti 2p AP-XPS data for samples prepared by TiO_2 ALD on SBA-15, taken under an atmosphere of 50 mTorr of H_2. The samples correspond to several numbers of TiO_2 ALD cycles, namely (from left to right): x = 2, 4, 6, 8, and 10. Three sets of data are shown in each panel, for three temperatures: 300 K (bottom, blue traces), 400 K (middle, purple), and 500 K (top, red).

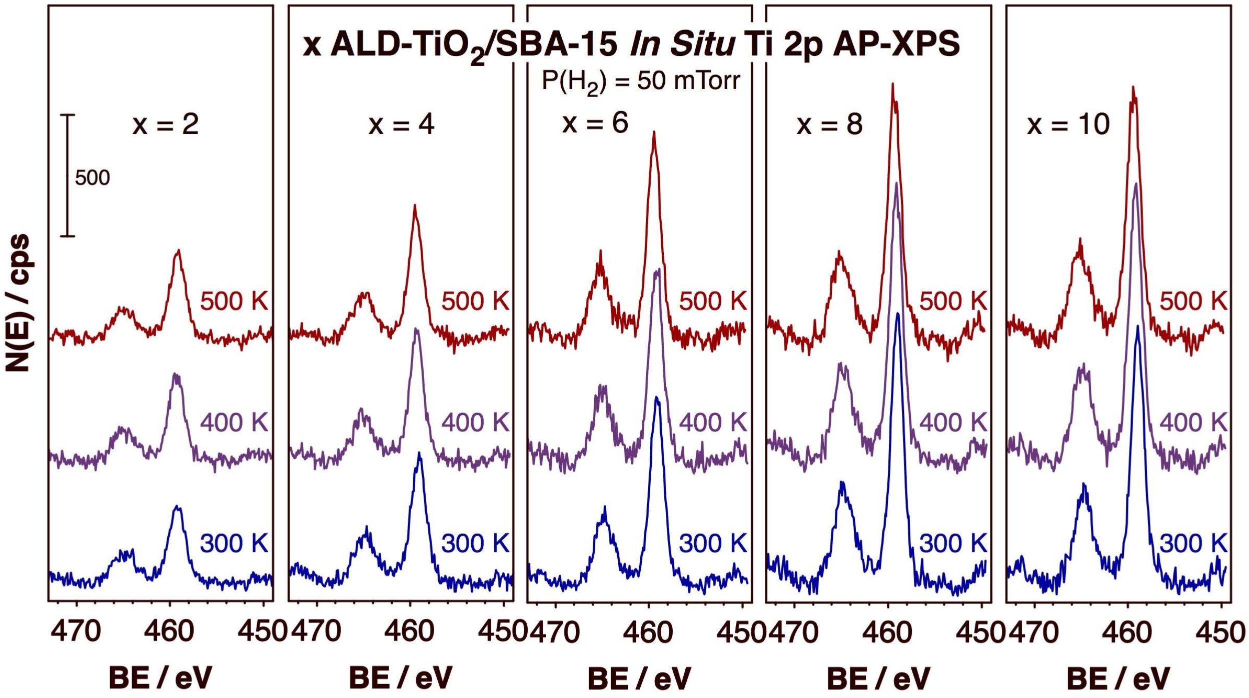

5. References

(1) Weng, Z.; Chen, Z.-h.; Qin, X.; Zaera, F. Sub-Monolayer Control of the Growth of Oxide Films on Mesoporous Materials. *J. Mater. Chem. A* **2018**, *6*, 17548-17558.

(2) Ke, W.; Liu, Y.; Wang, X.; Qin, X.; Chen, L.; Palomino, R. M.; Simonovis, J. P.; Lee, I.; Waluyo, I.; Rodriguez, J. A.et al. Nucleation and Initial Stages of Growth During the Atomic Layer Deposition of Titanium Oxide on Mesoporous Silica. *Nano Lett.* **2020**, *20*, 6884-6890.

(3) Cao, Y.; Chen, B.; Guerrero-Sánchez, J.; Lee, I.; Zhou, X.; Takeuchi, N.; Zaera, F. Controlling Selectivity in Unsaturated Aldehyde Hydrogenation Using Single-Site Alloy Catalysts. *ACS Catal.* **2019**, *9*, 9150-9157.

(4) *Handbook of X-Ray Photoelectron Spectroscopy*; Wagner, C. D.; Riggs, W. M.; Davis, L. E.; Moulder, J. F.; Muilenberg, G. E., Eds.; Perkin-Elmer Corporation: Eden Prairie, MN, 1978.
